# Periprosthetic fractures of the knee

**DOI:** 10.1530/EOR-2026-0075

**Published:** 2026-10-01

**Authors:** Kerstina Menzel, Carsten Perka, Panayiotis D Megaloikonomos, Henryk Haffer, Susana González Khatib, Clemens Gwinner, Emil Schemitsch, Sebastian Braun

**Affiliations:** ^1^ Center for Musculoskeletal Surgery, Charité – Universitätsmedizin Berlin, Corporate Member of Freie Universität Berlin and Humboldt-Universität Zu Berlin, Berlin, Germany; ^2^ First Department of Orthopedics, National and Kapodistrian University of Athens, Attikon University General Hospital, Athens, Greece; ^3^ Department of Surgery, Division of Orthopaedic Surgery, University of Western Ontario, London, Ontario, Canada

**Keywords:** knee arthroplasty, periprosthetic fracture, revision knee arthroplasty, osteosynthesis

## Abstract

- Periprosthetic fractures around total knee arthroplasty are an increasingly common and complex problem driven by rising arthroplasty volumes, aging populations, and longer implant survivorship. Successful management requires integration of trauma principles with arthroplasty-specific considerations, particularly implant stability, bone stock, and patient frailty.
- Implant stability and bone quality are the primary determinants of treatment strategy, outweighing fracture morphology alone. Classification systems are useful frameworks but must be interpreted in the context of component fixation and host factors.
- Fixation using locked plating, retrograde intramedullary nailing, or combined constructs is appropriate for stable implants with adequate bone stock and achieves high union rates, while preserving bone and future revision options, particularly important in younger or active patients.
- Revision arthroplasty with stemmed components, cones or sleeves, and appropriate constraint is indicated for loose components or compromised bone stock, offering durable reconstruction but with complication profiles similar to complex revision total knee arthroplasty.
- Distal femoral replacement serves as an effective salvage option for fractures that cannot be fixed or in frail, low-demand patients, enabling immediate weight-bearing but at the cost of higher complication and re-revision rates.
- Overall, outcomes depend not only on fracture union but also on restoration of alignment, extensor mechanism function, and early mobilization. High complication rates reflect the combined burden of fragility fracture biology and revision arthroplasty complexity, underscoring the need for individualized, patient-centered decision-making.

## Introduction

Periprosthetic fractures (PPFs) around total knee arthroplasty (TKA) represent a challenging complication with increasing clinical prevalence. As the number of primary and revision TKAs continues to rise worldwide, the absolute incidence of periprosthetic knee fractures has increased accordingly (1, 2, 3). Although relatively uncommon compared with other causes of TKA failure, these injuries are associated with substantial morbidity, prolonged rehabilitation, and increased mortality, particularly in older and frail patient populations (2, 4, 5).

The majority of PPFs around the knee occur in the distal femur and typically affect older patients with compromised bone quality following low-energy trauma (2, 6). In contrast, tibial and patellar PPFs are less frequent, but may pose equally complex reconstructive challenges, especially in the presence of component loosening or bone loss (6, 7). Importantly, fracture morphology alone rarely determines optimal management. Instead, implant stability, bone stock, and patient-related factors, such as functional demand and medical comorbidities, play a decisive role in treatment selection (2, 6, 8).

Several classification systems have been proposed to guide management of PPFs around the knee. The Su classification emphasizes the anatomical relationship of distal femoral fractures to the femoral component, whereas the Rorabeck classification focuses on implant stability and displacement. Tibial periprosthetic fractures are most commonly described using the Felix classification (7, 9, 10). While these systems provide useful frameworks, their clinical value lies primarily in supporting decision-making rather than dictating treatment.

Advances in fixation techniques and revision arthroplasty systems have expanded the range of available treatment options. Locked plating, retrograde intramedullary nailing, revision arthroplasty, and distal femoral replacement each have distinct indications, advantages, and limitations (8, 11, 12, 13, 14). Optimal outcomes depend not only on appropriate strategy selection but also on meticulous surgical technique and individualized consideration of patient frailty and rehabilitation potential.

Therefore, the aim of this review was to summarize the current evidence on epidemiology, classification, diagnostic evaluation, and contemporary management strategies for periprosthetic fractures around total knee arthroplasty, with particular emphasis on decision-making based on implant stability, bone stock, and patient-related factors.

## Epidemiology and risk factors

The incidence of PPFs around TKA has risen steadily over the past two decades, primarily due to increasing arthroplasty volume and improved implant survivorship (1, 2, 15). Most fractures occur several years after primary TKA, although intraoperative and early postoperative injuries also occur, often related to instrumentation during bone preparation or to postoperative falls (16). Registry data report an incidence of 0.3–5.5% following primary TKA, rising dramatically to up to 30% after revision TKA (1, 2, 6). Distal femoral fractures are the most common periprosthetic fractures after TKA, accounting for approximately 60–80% of all periprosthetic knee fractures and occurring in about 0.3–2.5% of patients after primary TKA. In comparison, periprosthetic fractures of the proximal tibia and patella are less frequent, with reported incidences of 0.4–1.7% and 0.2–0.9%, respectively (2, 6, 7). The substantially higher incidence after revision TKA reflects compromised bone stock, cumulative surgical trauma, and stress concentration around revision stems (6).

Patient factors play a dominant role. Advanced age, female sex, osteoporosis, chronic steroid use, neuromuscular disease, and frailty are strongly associated with fracture risk (3, 4, 6). Osteoporosis is present in up to 50% of affected patients and significantly delays fracture healing while increasing the risk of fixation failure and medical complications (2, 6). In older osteoporotic individuals, more than 90% of fractures result from low-energy trauma, such as a fall from a standing position, consistent with a fragility fracture mechanism. In contrast, younger patients with better bone quality more commonly sustain PPFs following high-energy trauma, including motor vehicle accidents or sports-related injuries (4, 5).

Implant-related factors that predispose to fracture include component design, fixation method, constraint, and prior revisions. Osteolysis, polyethylene wear, and structural bone loss contribute to weakening of the metaphysis and may precede a fracture (17, 18).

PPFs are associated with substantial morbidity and mortality. Reported one-year mortality rates range from 13 to 22%, comparable to those observed after hip fractures in frail older populations (4, 5). Postoperative complications, such as nonunion, malunion, infection, and thromboembolic events, are common and further complicate postoperative recovery (4, 5). Collectively, these data emphasize that PPFs of the knee in older patients represent a convergence of patient frailty, impaired bone quality, and mechanical vulnerability at the bone–implant interface, which significantly impacts early outcomes following these injuries. Conversely, younger patients with high functional expectations pose challenges related to durability of reconstruction and risk of future revision surgery (19).

### Classification

Classification systems aim to support decision-making by describing fracture location, implant stability, and bone quality (2, 20). Several classification systems have been proposed for PPFs around TKA. While these systems differ in anatomical focus and complexity, their primary value lies in structuring decision-making rather than dictating treatment. Among them, the Su classification and the Lewis–Rorabeck classification are most commonly applied for distal femoral fractures, whereas the Felix classification is used for tibial periprosthetic fractures (2, 6, 7, 9, 10).

The Lewis–Rorabeck classification (Table 1) emphasizes fracture displacement and femoral component stability, making it highly relevant for determining whether fixation or revision arthroplasty is required (10). It categorizes fractures into three groups: type I fractures are nondisplaced with a stable component and may be treated conservatively or operatively depending on patient factors, type II fractures are displaced but retain a stable implant and are typically managed with operative fixation, and type III fractures are defined by a loose femoral component and generally require reconstruction with revision arthroplasty.

**Table 1:** Lewis–Rorabeck classification (distal femur). The Lewis–Rorabeck classification categorizes distal femoral periprosthetic fractures according to the stability of the implant and displacement of the fracture.

Type	Definition	Implant	Strategy
I	Non-displaced	Stable	Non-operative vs fixation (ORIF)
II	Displaced	Stable	Fixation (LCP/RIMN)
III	± Displaced	Loose	Revision TKA vs DFR

ORIF, open reduction internal fixation; LCP, locked compression plate; RIMN, retrograde intramedullary nailing; TKA, total knee arthroplasty; DFR, distal femoral replacement.

The Lewis–Rorabeck classification categorizes distal femoral periprosthetic fractures according to the stability of the implant and displacement of the fracture.

The Su classification (Table 2) categorizes distal femoral PPFs based on their anatomical relationship to the femoral component, thereby providing valuable guidance for fixation strategies (9).

**Table 2:** Su classification (distal femur). The Su classification categorizes distal femoral periprosthetic fractures according to their relationship to the femoral component of a TKA.

Type	Definition	Implant	Strategy
I	Proximal to component	Stable	Fixation (LCP/RIMN)
II	At level of component	Stable	Fixation (LCP ± Adjuncts*)
III	Distal to flange (condylar)	Often unstable	Fixation vs revision TKA ± DFR (frail)

DFR, distal femoral replacement; LCP, locked compression plate; RIMN, retrograde intramedullary nailing; TKA, total knee arthroplasty.

* Adjuncts: possible nail, dual plating, or strut grafts.

Type I fractures occur proximal to the femoral component and usually allow standard fixation techniques. Type II fractures originate at the level of the femoral component and often require careful implant-sparing fixation strategies. Type III fractures extend distal to the anterior flange into the condylar or intercondylar region, where fixation options may be limited by reduced bone stock and proximity to the implant.

From a therapeutic perspective, the main strength of the Su classification lies in its ability to anticipate technical feasibility of fixation. Proximal and mid-level fractures (types I and II) are generally amenable to locked plating or retrograde intramedullary nailing, provided that the femoral component is stable and implant compatibility allows nail passage if nail is chosen (2, 9, 21). In contrast, type III fractures frequently challenge fixation due to limited distal bone stock and may necessitate revision arthroplasty or distal femoral replacement in selected patients, particularly when component stability or bone quality is compromised (2, 14).

The Su classification categorizes distal femoral periprosthetic fractures according to their relationship to the femoral component of a TKA.

The Felix classification (Table 3) addresses tibial PPFs and incorporates fracture location relative to the tibial keel or stem and implant stability (7). The Felix classification has four types of PPFs based on anatomical location: type I at the tibial plateau, type II adjacent to the tibial component, type III below the tibial component, and type IV representing intraoperative fractures that may occur at any location but commonly involve the tibial tubercle. Its main advantage is the explicit distinction between stable/well-fixed (subtype A) and loose (subtype B) components, which directly informs whether fixation or revision arthroplasty is required. Subtype C is used to describe intraoperative fractures.

**Table 3:** Felix classification (proximal tibia). The Felix classification classifies periprosthetic tibial fractures according to their location and the stability of the component.

Type	Fracture location/subtype	Implant stability/timing	Treatment implication
I	Tibial plateau		
	A	Stable	Fixation if displaced; non-operative if stable/aligned
	B	Loose	Revision TKA (stemmed tibial component)
	C	Intraoperative	Immediate fixation ± stem
II	Adjacent to tibial stem or keel		
	A	Stable	Fixation (plate/cables), protect keel
	B	Loose	Revision TKA (stem ± cone/sleeve)
	C	Intraoperative	Fixation ± conversion to stem
III	Distal to tibial component (shaft region)		
	A	Stable	Treat as tibial shaft fracture (IMN or plate)
	B	Loose	Revision TKA ± shaft fixation
	C	Intraoperative	Fixation during index procedure (fixation ± conversion to stem)
IV	Intraoperative fracture (any location, often tibial tubercle)		
	-	Intraoperative	Immediate fixation ± stem

TKA, total knee arthroplasty; IMN, intramedullary nailing.

The Felix classification classifies periprosthetic tibial fractures according to their location and the stability of the component.

Across systems, a fundamental principle persists: implant stability and bone stock are the principal determinants of management (1, 2, 10). Ultimately, classification systems should be regarded as decision-support tools that must be integrated with consideration of patient frailty, bone quality, and functional demands to guide definitive treatment (2, 6, 8).

In addition to joint-specific classification systems, the Unified Classification System (UCS) has been proposed as a comprehensive framework applicable to PPFs throughout the musculoskeletal system. The UCS expands the principles of the Vancouver classification and categorizes fractures according to their anatomical relationship to the implant and surrounding bone. Six fracture types are described: type A (apophyseal fractures involving tendon or ligament attachments), type B (fractures at the level of the implant bed, further subdivided into B1–B3 depending on implant stability and bone quality), type C (fractures distant from the implant), type D (fractures occurring between two prostheses within the same bone), type E (fractures involving two bones supporting a single prosthesis), and type F (fractures of an articular surface articulating with an implant). Although not specific to knee arthroplasty, the UCS provides a useful conceptual framework for understanding complex periprosthetic fracture patterns and guiding treatment principles across different joints. Therefore, it cannot replace the classification specific for PPFs of the knee but can be used as an additional tool to help understand the fractures.

More recently, Kim *et al.* have proposed a classification system specifically designed for periprosthetic distal femoral fractures treated with locked plating after TKA, aiming to improve surgical decision-making in complex fixation scenarios (22). In contrast to purely morphology- or stability-based systems, this classification emphasizes factors directly influencing fixation strategy, including the quality of the distal bone segment, medial cortical support, and the feasibility of achieving stable fixation with a lateral locked plate alone. The classification describes three types of fractures: type I fractures (main fracture line proximal to the anterior flange of the femoral component) should be treated with a single lateral locking plate, type II fractures (main fracture line distal to the anterior flange of the femoral component) should be treated with dual plating, and type III fractures (component loosening or instability regardless of the fracture line) are recommended for a revision arthroplasty (22).

### Diagnosis

The first step of the diagnostic algorithm is establishing the mechanism of injury, timing relative to arthroplasty, and the presence of prodromal symptoms, such as pain, instability, or reduced function, which may indicate pre-existing implant loosening or osteolysis (1, 2). Most fractures result from low-energy falls in older individuals, whereas high-energy trauma is more typical in younger patients.

Clinical examination must assess limb alignment, neurovascular status, and soft-tissue integrity, particularly the extensor mechanism. Painful pre-injury ambulation, instability, or progressive varus/valgus deformity may suggest component loosening (23).

### Imaging

Standard radiographs of the entire femur and tibia with standard anteroposterior (AP) and lateral views are essential to evaluate fracture pattern, component positioning, bone stock, and loosening. Full-length imaging is recommended to identify interprosthetic scenarios and malalignment (24). Computed tomography (CT) improves delineation of fracture morphology, implant–bone interface, bone stock, and osteolysis when standard radiographs are insufficient (25). When patellar fractures or extensor mechanism injury are suspected, skyline views and ultrasound/magnetic resonance imaging (MRI) may assist.

## Laboratory evaluation

As periprosthetic joint infection (PJI) may mimic loosening or osteolysis, inflammatory markers (erythrocyte sedimentation rate (ESR) and C-reactive protein (CRP)) and, when indicated, aspiration should be considered, especially in painful pre-injury knees or in cases planned for revision surgery (26).

## Key determinants of management

Preoperative evaluation must answer three essential questions:

- Is the implant stable?
- Is bone stock adequate for fixation (ORIF) or revision arthroplasty?
- What is the patient’s physiological and functional profile?

These questions drive treatment selection and have greater prognostic value than fracture pattern alone (2, 8, 23).

### Treatment strategies

Management of PPFs around TKA must integrate surgical feasibility, implant stability, and patient physiology. Three principal strategies exist along a continuum: fixation/ORIF, revision arthroplasty, and distal femoral replacement (DFR). Procedure selection should take biological healing potential, implant longevity, and rehabilitation demands into account (2, 8). Classification systems provide a structured framework to guide treatment selection, but should be applied in a complementary fashion rather than in isolation (2, 6, 8).

Successful management of PPFs after TKA requires meticulous preoperative planning and adherence to several key technical principles (2, 6). Assessing component stability accurately is essential, as treatment selection depends more on implant fixation and bone stock than on fracture morphology (2, 8, 10). CT can help clarify doubtful bone–implant interfaces (25). Early mobilization should be prioritized whenever possible, especially in frail older patients, as prolonged protected weight-bearing significantly increases medical morbidity (4, 5).

Non-operative management may be considered in carefully selected patients with Rorabeck type I distal femoral fractures or Felix type IA tibial fractures, provided that the component remains stable, fracture displacement is minimal or absent, and overall alignment is acceptable (7, 8, 10). Typical treatment protocols include hinged knee bracing or immobilization, protected or partial weight-bearing, and close serial radiographic follow-up to monitor for secondary displacement or implant loosening (2, 8, 27). Early mobilization within protected limits is generally encouraged to reduce complications associated with prolonged immobilization in older patients. Reported outcomes are variable but may be satisfactory in low-demand patients with stable fracture patterns and reliable compliance, although prolonged healing times, secondary displacement, and loss of function remain recognized risks (2, 8, 10). Consequently, non-operative treatment is usually reserved for non-displaced fractures with preserved implant fixation or for patients who are medically unfit for surgery.

### Fixation/ORIF

Fixation is preferred when the component is stable and metaphyseal bone stock is adequate for union. This scenario is most common in displaced supracondylar fractures of the distal femur with stable components. Fixation is primarily indicated in Su type I and II, Rorabeck I and II, and Felix type A and C fractures. Locked compression plating (LCP) and retrograde intramedullary nailing (RIMN) represent the two most commonly used fixation strategies for periprosthetic distal femoral fractures with a stable femoral component. A minimally invasive lateral locking plate construct is illustrated in Figs 1 and 2. LCP provides angular stability and polyaxial screw options suitable for osteoporotic bone (11, 12). The fracture pattern is a critical determinant when selecting the optimal fixation strategy for periprosthetic distal femoral fractures. In particular, fractures with significant metaphyseal or diaphyseal–metaphyseal comminution and loss of medial cortical support present a substantial mechanical challenge for lateral plate fixation alone (28). When the medial column is deficient, a single lateral locked plate functions as a long cantilever construct and must resist varus forces almost entirely on its own, which increases plate bending stresses and may result in progressive varus collapse, loss of reduction, and eventual fixation failure or nonunion. In such fracture patterns, reconstruction of the medial column becomes essential. This can be achieved either through medial buttress plating or through dual plating constructs that supplement the lateral locked plate. Dual plating consists of a standard lateral distal femoral locking plate combined with an additional medial plate, most commonly a small fragment 3.5 mm LCP. Although larger fragment plates or anatomical medial distal femoral plates may be required in extensive metaphyseal deficiency or osteoporotic bone (8, 11, 28, 29, 30), the medial plate should be of sufficient length to provide metaphyseal and supracondylar support, ideally allowing fixation with at least three bicortical screws on either side of the fracture where feasible, while acting as a medial buttress to enhance varus stability rather than spanning the entire femoral diaphysis. The lateral plate should generally span the majority of the femoral diaphysis to reduce stress concentration. Usually, the lateral plate is applied first to restore alignment and length. Medial plate insertion may be performed through a separate direct medial or subvastus approach, while minimal invasive or percutaneous techniques can reduce additional soft-tissue trauma (29, 30). An example of dual plating following failed fixation with an isolated lateral locking plate is shown in Fig. 3. In most contemporary techniques, the second plate is placed medially to restore medial cortical support and avoid interference with the patellofemoral joint. Alternatively, the medial plate may be contoured into a helical configuration to facilitate minimally invasive insertion, improve access to the distal femur while avoiding the medial neurovascular structures, and provide stable medial support; however, despite encouraging biomechanical and early clinical results, evidence for its use in periprosthetic distal femoral fractures remains limited (31, 32). While orthogonal ‘90–90’ constructs have historically been described, dual plating constructs are more commonly used in distal femoral PPFs because the anterior flange of the femoral component and the typical metaphyseal defect above it often limit safe anterior plate placement. Alternatively, cortical strut allografts may be used to recreate medial support and provide biological augmentation in selected cases. These strategies redistribute mechanical loads, improve resistance to varus collapse and rotational forces, and are particularly relevant in osteoporotic bone, short distal segments, and fractures with extensive metaphyseal comminution where isolated lateral plating is unlikely to provide sufficient stability. From a surgical planning perspective, the indication for additional medial fixation is primarily determined by the degree of metaphyseal comminution, loss of medial cortical continuity, short distal segments, and concern for varus collapse or nonunion (28, 29). Biomechanically, dual-plate constructs may provide improved stability compared with isolated lateral locked plating, particularly in osteoporotic bone and complex fracture patterns, although they require greater soft-tissue dissection and increased surgical complexity (8, 29). The classification by Kim *et al.* can be used for surgical decision-making (22).

**Figure 1:**
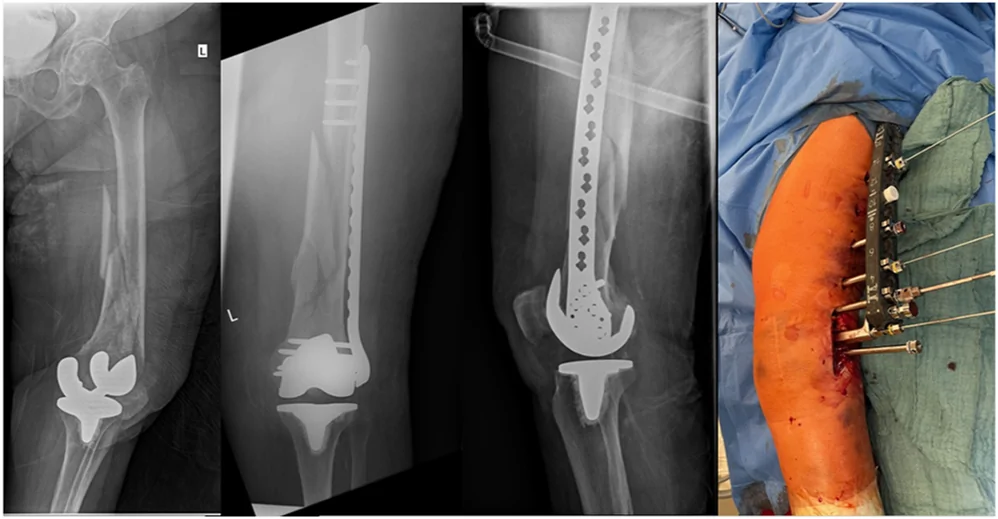
Periprosthetic distal femoral fracture treated with lateral locking plate osteosynthesis. (A) Preoperative anteroposterior (AP) radiograph demonstrating a periprosthetic femoral fracture around a well-fixed total knee arthroplasty, classified as Lewis–Rorabeck type I and Su type I. (B) Postoperative AP radiograph following open reduction and internal fixation (ORIF). (C) Postoperative lateral radiograph demonstrating fixation with a lateral locking plate. (D) Intraoperative image illustrating the minimally invasive plate osteosynthesis (MIPO) technique.

**Figure 2:**
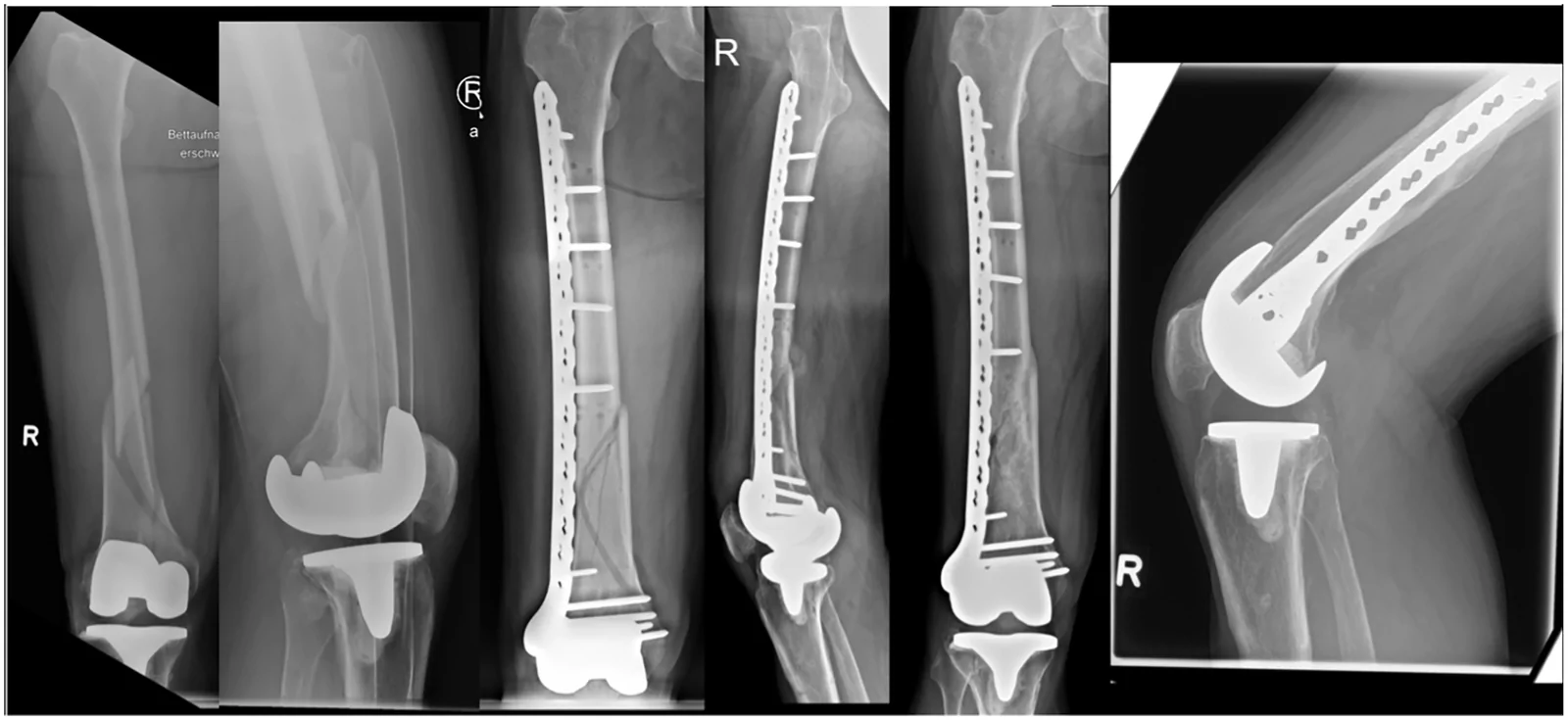
Radiographic course following dual plating for periprosthetic fracture treatment. Preoperative radiographs of the periprosthetic fracture in anteroposterior (A) and lateral (B) views, radiographs at 6-week postoperative follow-up following dual plating fixation in anteroposterior (C) and lateral (D) views, and radiographs at 1-year follow-up in anteroposterior (E) and lateral (F) views.

**Figure 3:**
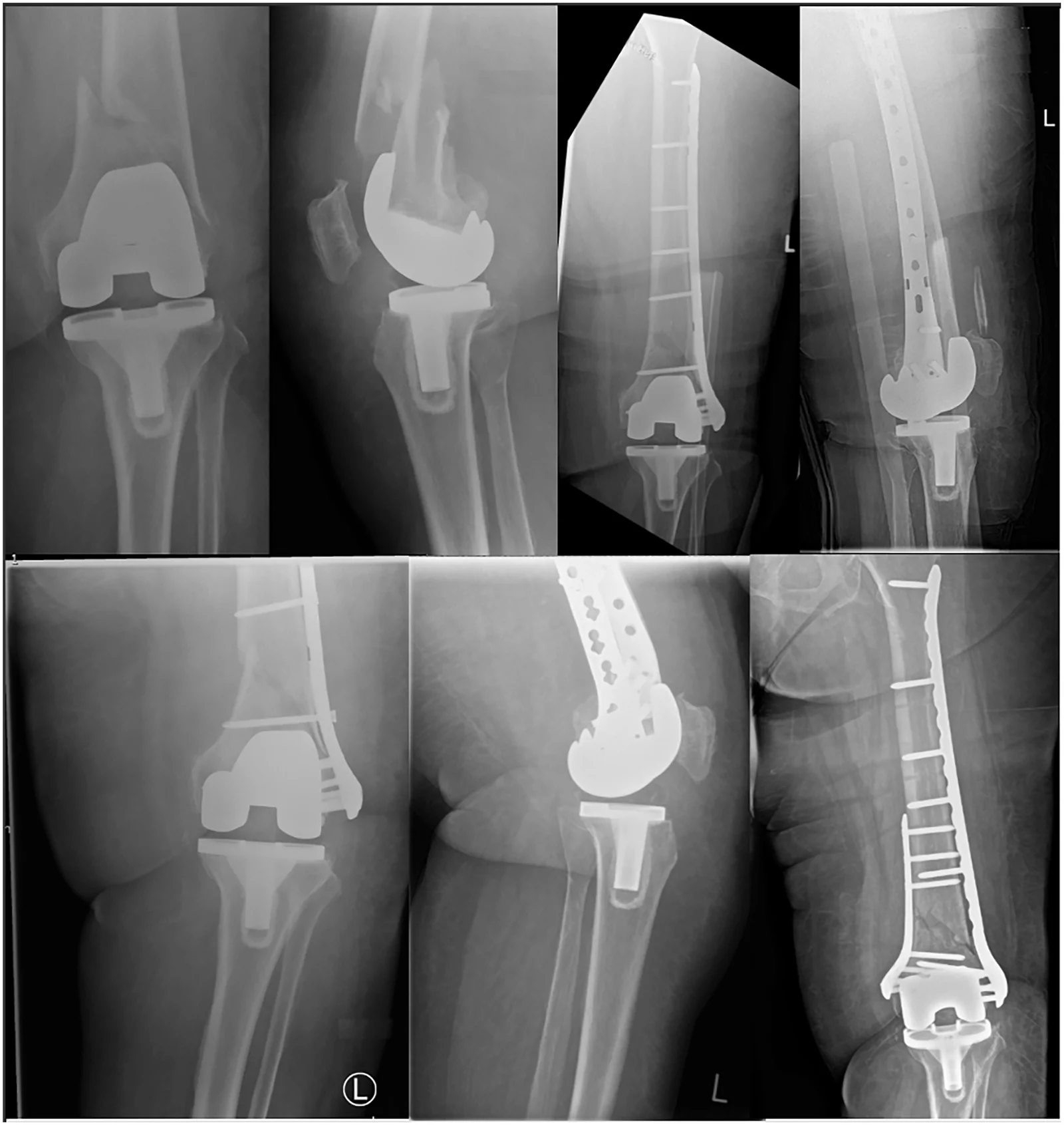
Radiographic sequence of periprosthetic fracture treatment and revision. Preoperative radiographs of the periprosthetic fracture in anteroposterior (A) and lateral (B) views, postoperative radiographs following fixation with a lateral locking plate in anteroposterior (C) and lateral (D) views, postoperative complication demonstrating medial varus collapse (E), and radiographs after revision surgery with dual plating in lateral (F) anteroposterior (G) views.

### Technical notes

- Use long plates with sufficient working length to avoid stress concentration (MIPO techniques may help not to do extended soft-tissue releases).
- Polyaxial locking screws facilitate screw placement around the prosthesis.
- Aim for relative stability to promote callus formation.
- Avoid short plates and excessive stiffness, which increase nonunion risk.

RIMN represents an alternative fixation strategy for periprosthetic distal femoral fractures when the femoral component design allows nail passage through the intercondylar box (21). When feasible, RIMN provides union rates comparable to locked plating (approximately 85–95%) and offers biomechanical advantages through load-sharing fixation, which may reduce implant stresses and facilitate earlier mobilization (21). However, its applicability depends strongly on implant compatibility; therefore, knowledge of the specific arthroplasty design, manufacturer, and component size is essential during preoperative planning to confirm adequate intercondylar box dimensions and safe nail trajectory. Several studies have reported the notch dimensions and clearance distances for commonly used femoral components, which can help determine whether retrograde nail insertion is technically feasible (33, 34, 35).

Nail fixation is particularly attractive for fractures with sufficient distal bone stock that permit multiple distal interlocking screws, allowing stable fixation below the femoral component. In contrast, very distal fractures, severe metaphyseal comminution, or limited distal fixation options may compromise the stability of an isolated nail construct (29). In these scenarios, augmentation strategies, such as nail–plate combination constructs, have been increasingly described. Nail–plate combination constructs are particularly useful in osteoporotic bone, highly comminuted fractures, or very short distal segments where enhanced load-sharing and immediate weight-bearing are desired (29, 30, 36). In these constructs, a lateral locking plate is combined with a retrograde nail to enhance axial and rotational stability, particularly in osteoporotic bone, highly comminuted fractures, or fractures with short distal segments where either fixation method alone may be insufficient (37). Compared with dual plating strategies, nail–plate constructs provide additional load-sharing through the intramedullary device while the plate improves rotational control and supports distal fixation (36).

Technical considerations are critical for successful retrograde nailing. The entry point must be carefully chosen within the intercondylar notch to avoid malreduction or displacement of the distal fragment. Incorrect entry points may lead to extension deformity or malalignment, particularly in short distal fragments. In addition, the proximal curvature and diameter of the nail must match the femoral anatomy to prevent cortical impingement or iatrogenic fracture. With careful implant selection, meticulous technique, and appropriate patient selection, RIMN, either alone or combined with plate fixation, can allow stable fixation and facilitate early weight-bearing, which is increasingly prioritized in older patients to reduce medical complications and functional decline (29, 38).

### Technical notes

Reduction of PPFs around the knee may be challenging due to deforming muscular forces acting on the distal fragment. The gastrocnemius muscle may pull the distal femoral fragment posteriorly, leading to flexion deformity, while the adductor muscles may contribute to varus alignment. In open approaches with plate fixation, partial release of the gastrocnemius or direct manipulation of the fragment can facilitate reduction. However, in closed techniques such as RIMN, this is usually not possible, and surgeons must rely on indirect reduction maneuvers to counteract these competing forces.

- Confirm intercondylar box compatibility preoperatively.
- Ensure adequate distal fixation (multiple locking options).
- Consider nail–plate combinations in osteoporotic or comminuted fractures.

In addition to multiple multiplanar distal locking screws, the use of end-cap locking nuts or washers may enhance screw–nail interface stability and reduce the risk of screw backout or distal loosening. Furthermore, newer systems allow the addition of a medial locking attachment plate or washer locking to the nail, thereby increasing rotational stability and resistance to varus or valgus collapse in highly comminuted fractures or limited distal bone stock (33, 34, 35, 36).

Nonunion (6–18%), malalignment (10–20%), and fixation failure and reoperation (10–25%) are the most frequent surgical complications (11, 12, 38). Infection rates are relatively low (2–6%). Frail patients remain vulnerable to medical complications, including pneumonia, thromboembolism, and loss of independence, with a reported 1-year mortality of 4–11% in older cohorts (5). The comparatively lower mortality reported after fixation is likely influenced by selection bias, as healthier patients are more likely to be selected for fixation than the overall PPF population.

Comparative studies and systematic reviews have demonstrated similar union rates for both plating and nailing techniques, generally ranging between 80 and 95%, with no clear superiority of one method in terms of fracture healing (11, 12, 21). LCP offers greater versatility in complex fracture patterns, particularly in very distal fractures or in the presence of poor bone stock, as polyaxial locking screws allow implant-sparing fixation around the femoral component (8, 11, 12). In contrast, RIMN provides a biomechanical advantage through load-sharing fixation, potentially facilitating earlier weight-bearing and reducing construct stiffness, but its applicability is limited by femoral component design, intercondylar box compatibility, and the ability to achieve adequate distal fixation (21). Malalignment and distal fixation failure remain recognized risks with RIMN in osteoporotic bone or short distal segments (21, 37). In contrast, recent clinical series by Wall *et al.* demonstrated comparable functional outcomes between plating and nailing strategies, while Abboud *et al.* reported that RIMN may permit faster recovery of weight-bearing capacity and improved early Parker mobility scores in older patients, without clear differences in mortality rates (39, 40). Recent comparative studies and meta-analyses demonstrated largely equivalent union and complication rates between contemporary RIMN and locked plating constructs for supracondylar PPFs, without definitive evidence supporting universal superiority of either strategy (29, 39, 40 41, 42). However, RIMN may facilitate earlier weight-bearing and functional recovery in selected older patients due to its load-sharing biomechanics, whereas locked plating remains advantageous in very distal fracture patterns or when nail compatibility is limited (29, 33, 34, 35, 40).

### Revision arthroplasty

Revision arthroplasty is indicated in Rorabeck type III, Su type III, and Felix type B fractures when components are loose, bone stock is compromised, or fixation is unlikely to provide durable stability. The intent is to restore the bone–implant interface, alignment, and ligamentous balance.

Stemmed components bypass fracture regions and provide diaphyseal engagement. However, successful revision arthroplasty requires sufficient metaphyseal support, ideally with remaining zone 1 bone or at least a supportive zone 2, which can be augmented using porous metal cones or sleeves (43, 44). Diaphyseal fixation alone (zone 3) is typically insufficient to provide durable support in these situations; if adequate metaphyseal support cannot be obtained, DFR should be considered instead. Constraint selection reflects ligament integrity: varus–valgus constrained implants may be required when collateral ligaments are compromised, whereas rotating-hinge designs are reserved for cases with severe instability or major bone loss (45). Importantly, ligament competence is largely determined by fracture involvement of the collateral ligament insertions at the femoral condyles, particularly the medial and lateral collateral ligaments.

Medium-term survivorship for revision TKA performed in this setting ranges from 78 to 90% at 5 years (13, 43, 44, 45). Functional improvement is typically moderate and depends on preexisting pathology and functional status of the patient.

Complications resemble those of revision TKA generally, including aseptic loosening (5–10%), stiffness (5–12%), infection (4–10%), and instability (13, 43, 44, 45). Extensor mechanism issues may emerge over time, influencing gait and endurance.

Revision arthroplasty versus DFR is favored in younger, active patients, or those with sufficient bone stock to warrant the need for long-term implant durability.

### Technical notes

- Use stemmed components (preferably cementless) to bypass fracture zones and restore alignment.
- Address metaphyseal bone loss and support revision construct with cones or sleeves.
- Match implant constraint to ligament competence.

### Distal femoral replacement

DFR is reserved for Su type III fractures with non-reconstructable fractures, catastrophic bone loss, failed fixation or revision (see Fig. 4), and older or frail individuals for whom prolonged protected weight-bearing is unrealistic (14).

**Figure 4:**
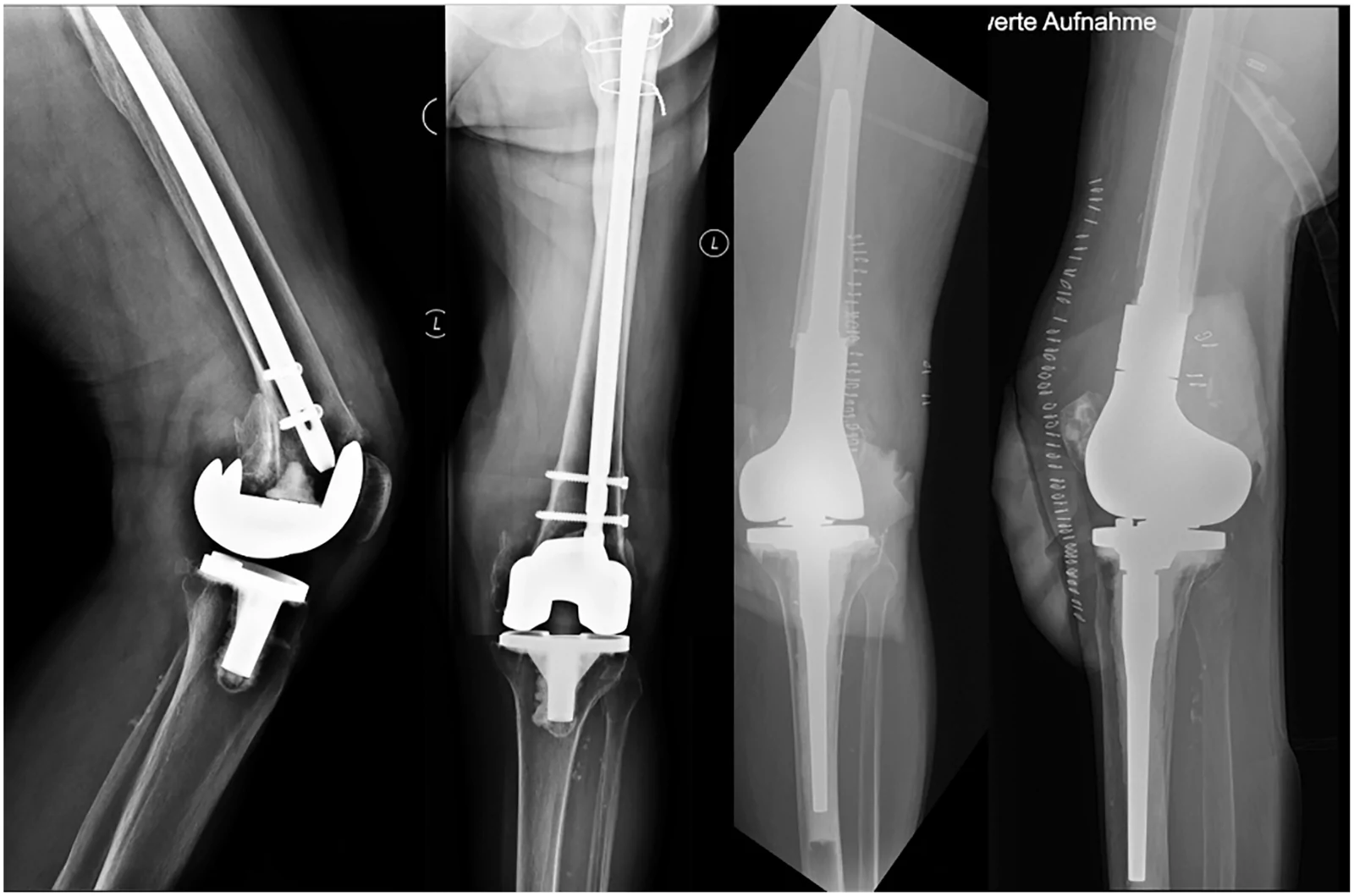
Interprosthetic distal femoral fracture treated with distal femoral replacement. (A) Preoperative AP radiograph demonstrating an interprosthetic distal femoral fracture located between a long cephalomedullary nail used for prior subtrochanteric femoral fracture fixation and a total knee arthroplasty. (B) Preoperative lateral radiograph of the same fracture. (C) Postoperative AP radiograph following conversion to a cemented distal femoral replacement. (D) Postoperative lateral radiograph confirming implant position and reconstruction.

DFR substitutes the distal femur, restores alignment, and allows immediate full weight-bearing. Operative time is often shorter than complex revision or complex dual fixation procedures, which may reduce perioperative morbidity.

Short-term survivorship is favorable (85–95% at 1 year), but durability declines beyond 5 years, with survivorship being 55–75% at mid- to long-term follow-up (14, 46). With regard to fixation technique, both cemented and cementless stems are currently used in DFR although there are no studies specifically comparing these fixation strategies exclusively in the setting of PPFs. Most available data include mixed indications, such as tumor reconstruction, aseptic loosening, infection, and fractures. In a recent cohort study evaluating DFR survivorship across fixation methods, no significant difference in revision rates or causes of failure was observed between cemented and cementless constructs, with a reported 5-year survivorship of approximately 72% for cemented and 87% for cementless stems, although these differences were not statistically significant (47).

DFR carries the highest complication burden. Re-revision rates of 18–32% are reported at mid-term follow-up, predominantly due to aseptic loosening (8–20%), infection (8–15%), and instability (6–14%) (14, 46). Extensor mechanism failure occurs in 4–12% and is functionally devastating (48).

In clinical practice, cemented stems are often favored in older patients with poor bone quality, as they provide immediate fixation and facilitate early weight-bearing. This aligns with the typical indication for DFR, which is most appropriate for low-demand, older, or medically frail patients requiring rapid mobilization and reliable pain relief rather than long-term durability. Cementless fixation, in contrast, may offer theoretical advantages in younger patients with better diaphyseal bone stock by allowing biological fixation and potentially reducing the risk of long-term aseptic loosening. Nevertheless, current evidence suggests that implant survival is likely influenced more by patient factors, indication, and the extent of bone loss than by fixation method alone.

### Technical notes

- Ideal for older, frail, low-demand patients.
- Protection of the extensor mechanism is essential.
- Avoid DFR in younger or active patients when reconstructive or fixation options exist.

A pragmatic algorithm synthesizes the above; see Fig. 5.

**Figure 5:**
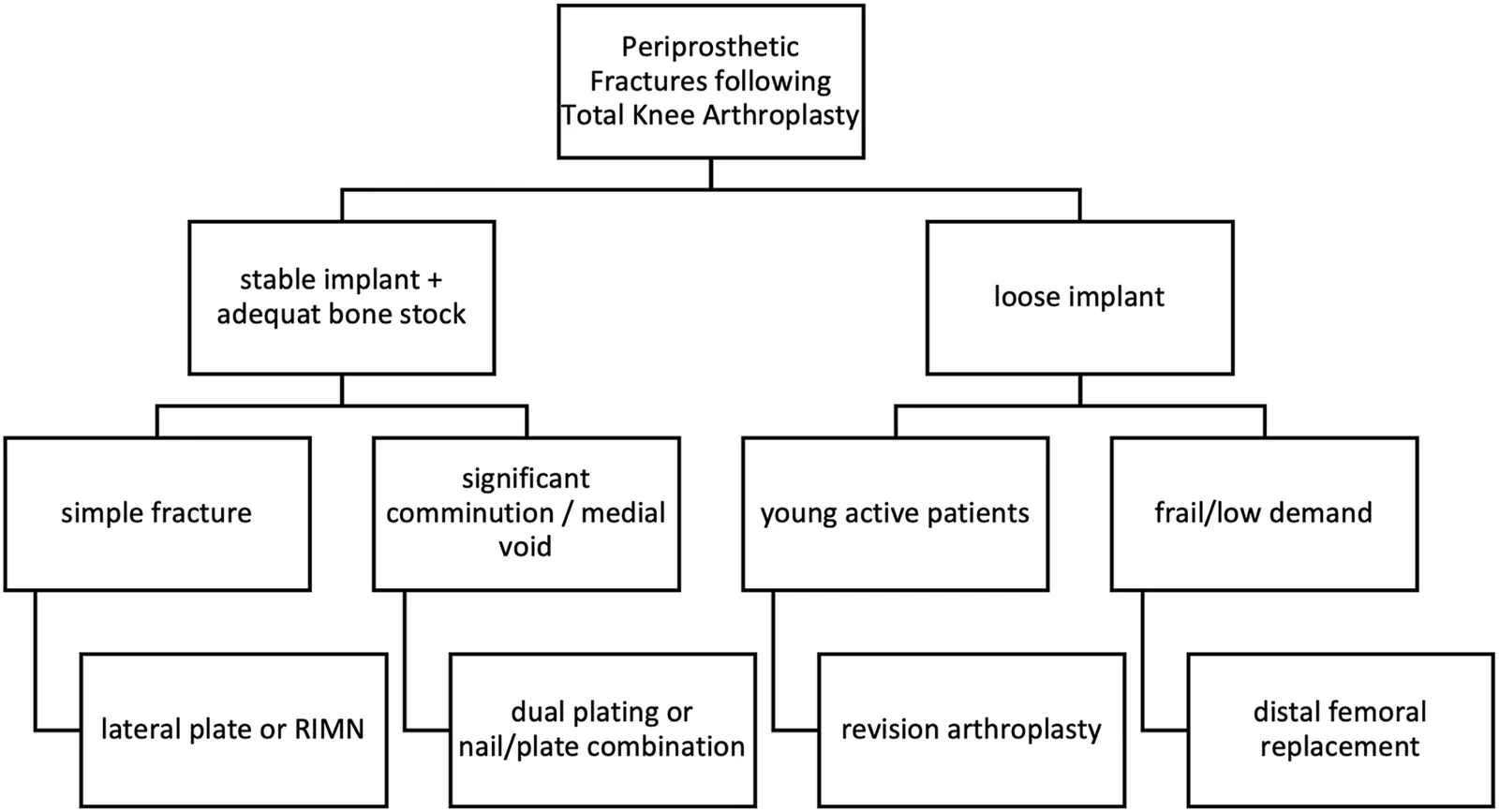
Algorithm depicting the treatment decision-making for periprosthetic distal femur fractures following total knee arthroplasty.

### Treatment of proximal tibial PPFs

When the tibial component is stable and bone stock is preserved, operative fixation is the preferred strategy for displaced fractures or fractures at risk of secondary displacement. Depending on fracture location and implant geometry, fixation options include locking plate osteosynthesis, minimally invasive bridge plating, lag screw augmentation, or in selected distal fractures, intramedullary fixation if the prosthetic stem does not obstruct the canal (7, 27, 49). Locked plating is particularly useful for metaphyseal and peri-implant fractures because polyaxial screw trajectories may facilitate fixation around a tibial keel or stem. Technical principles include use of long bridging constructs, preservation of periosteal blood supply, and restoration of mechanical alignment. Complications after fixation include delayed union, nonunion, malalignment, and wound problems, particularly in osteoporotic bone or highly comminuted patterns (27, 49).

Fractures associated with a loose tibial component (Felix type B) generally require revision TKA, as isolated fracture fixation without addressing implant loosening is associated with mechanical failure and persistent pain (7, 13, 50). Revision strategies typically involve stemmed tibial components that bypass the fracture zone, combined with metaphyseal fixation using cones or sleeves when bone loss is present (50). Supplemental plate fixation or cerclage may be necessary when fracture stability cannot be adequately controlled by the revision construct alone. In younger or higher-demand patients, revision arthroplasty aims to restore durable fixation and limb alignment while preserving bone stock for future procedures (13, 50).

In cases of severe bone loss, non-reconstructable fractures, or frail low-demand patients, salvage strategies, such as megaprosthetic reconstruction or proximal tibial replacement, may be considered, although these remain uncommon and are associated with substantial complication rates, including infection, extensor mechanism dysfunction, and aseptic loosening (14, 46). Therefore, such options are generally reserved for exceptional circumstances.

Overall, outcomes of tibial PPF treatment are less well studied than distal femoral fractures, but available evidence suggests that successful results depend predominantly on accurate recognition of implant stability and selection of a mechanically robust construct. Stable implants treated with appropriate fixation generally heal reliably, whereas fractures associated with loosening or major bone deficiency have higher rates of reoperation and functional compromise (7, 27, 49). Consequently, a classification-driven treatment algorithm integrating implant fixation, bone stock, and patient frailty is recommended for optimal decision-making.

### Periprosthetic patellar fractures

Periprosthetic patellar fractures (PPPFs) represent the least common form of PPFs around TKA, with reported incidences ranging from 0.2 to 0.9% after primary TKA, and substantially higher rates following revision procedures (51, 52). Despite their low frequency, these injuries pose significant therapeutic challenges and are associated with high complication rates, particularly when surgical intervention is required (27, 52).

These fractures may result from direct trauma, such as a fall onto the anterior knee, but more commonly arise from non-traumatic mechanisms related to surgical technique, implant design, or compromised patellar vascularity (49, 51, 52). Extensive soft-tissue dissection, medial parapatellar arthrotomy, lateral retinacular release, patella resurfacing, and revision surgery have been shown to impair patellar blood supply, predisposing the patella to insufficiency fractures and osteonecrosis (49, 51). Additional risk factors include osteoporosis, obesity, inflammatory arthritis, excessive knee flexion, malalignment, and reduced residual patellar bone stock (27, 52).

Several classification systems for PPPFs have been described; however, the classification proposed by Ortiguera and Berry is most widely used and clinically relevant (51). This system emphasizes three key determinants that directly influence treatment decisions: integrity of the extensor mechanism, stability of the patellar component, and quality of remaining patellar bone stock. Type I fractures are characterized by an intact extensor mechanism and a stable patellar component. Type II fractures involve disruption of the extensor mechanism, regardless of implant stability. Type III fractures are defined by a loose patellar component and are further subdivided based on residual bone stock (type IIIA: adequate bone stock; type IIIB: poor bone stock) (51, 52).

Treatment strategies are primarily dictated by those three key determinants listed above rather than fracture morphology alone; see Fig. 6. Nonoperative management is generally recommended for type I fractures, as several studies have demonstrated satisfactory pain relief and functional outcomes with immobilization and protected mobilization when both the extensor mechanism and implant remain intact (51, 52). In contrast, operative treatment of PPPFs is associated with high complication rates, including nonunion, infection, extensor lag, and reoperation (27, 52).

**Figure 6:**
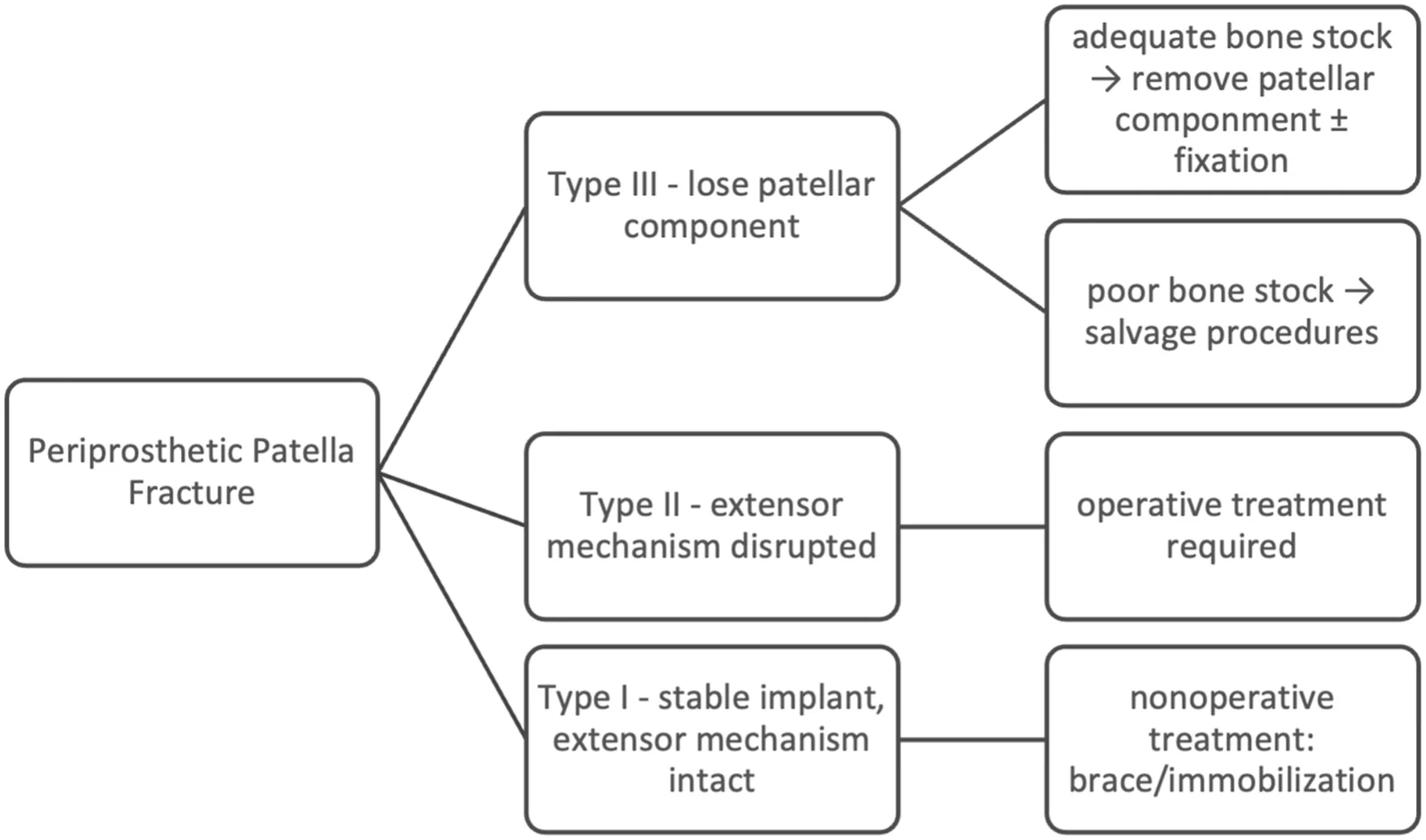
Flow chart demonstrating the treatment decision algorithm for periprosthetic patella fractures.

Type II fractures typically require surgical intervention because extensor mechanism disruption compromises knee function and prosthesis longevity (27, 51). Surgical options include open reduction and internal fixation (ORIF), partial patellectomy with tendon repair, or extensor mechanism reconstruction, depending on fracture pattern and bone quality. Even with operative treatment, reported outcomes remain guarded, with substantial rates of persistent extensor lag and reoperation (27, 52).

Management of type III fractures remains particularly challenging. In cases with adequate residual bone stock (type IIIA), removal of the patellar component with or without fixation may be considered, as revision of the patellar component is generally discouraged due to limited fixation potential and high failure rates (51, 53). In the presence of poor bone stock (type IIIB), treatment options are limited and may include patellar component excision, patellectomy, or extensor mechanism reconstruction using allograft tissue (51, 52, 53). While these salvage procedures may provide pain relief, they are frequently associated with inferior functional outcomes (52, 53).

Overall, current evidence consistently suggests that nonoperative treatment yields the most reliable results whenever feasible, and surgical intervention should be reserved for cases with extensor mechanism failure or symptomatic implant loosening, with careful patient selection and realistic expectations regarding outcomes (27, 51, 52).

## Future directions

Future developments in the management of PPFs after TKA are likely to focus on improving implant compatibility with fixation strategies, expanding reconstruction options for bone loss, and enhancing rehabilitation pathways. Modern revision implants increasingly incorporate open intercondylar boxes that facilitate retrograde nail passage, broadening the indications for load-sharing constructs in younger and active patients (54). Advances in porous metal technology, particularly cones and sleeves, have improved osseointegration and may reduce the need for salvage procedures in the presence of severe metaphyseal defects (50).

On the biological side, enhanced fixation strategies for osteoporotic bone, including augmented plating, cement augmentation, and hybrid constructs, continue to evolve (55). Early mobilization protocols and multidisciplinary perioperative care have gained importance, particularly in frail older populations in whom medical morbidity and functional decline remain substantial drivers of outcome (5).

Finally, improved registry data and international collaboration are needed to refine indications for DFR versus revision arthroplasty, as durability and re-revision risks remain incompletely understood. Patient-reported outcomes should complement survivorship metrics to better capture functional recovery and quality of life.

### Key take-home messages

- Implant stability and bone stock are the principal determinants of treatment selection.
- Fixation offers high union rates and preserves bone stock when components are stable.
- Revision arthroplasty provides durable reconstruction for loose components or bone loss.
- Distal femoral replacement remains a valuable salvage option for frail, older, or low-demand patients.
- Complication rates are high and reflect the dual burden of fragility fracture biology and revision arthroplasty complexity.
- Procedure selection must consider patient frailty, life expectancy, and rehabilitation capacity as strongly as fracture morphology.

## ICMJE Statement of Interest

C Perka reports royalties from Zimmer, Johnson & Johnson MedTech, and Smith & Nephew and consulting fees from Zimmer and Johnson & Johnson MedTech. E Schemitsch reports royalties from Amgen Canada, Smith & Nephew, and Stryker Corporation. PAll other authors report no conflicts of interest.

## Funding Statement

This work did not receive any specific grant from any funding agency in the public, commercial, or not-for-profit sector.

## Author contribution statement

KM contributed to data acquisition, literature review, and manuscript drafting. CP contributed to study conception and supervision, critical revision of the manuscript, and final approval of the submitted version. PDM contributed to literature review, data interpretation, and critical manuscript revision. HH contributed to literature review, data interpretation, and critical manuscript revision. SGK contributed to literature review, data interpretation, and critical manuscript revision. CG contributed to literature review, data interpretation, and critical manuscript revision. ES contributed to expert input, critical appraisal of the content, and manuscript revision. SB contributed to study conception and design, critical analysis and interpretation of the literature, and manuscript drafting and revision.
